# Analysis of glutamate-dependent mechanism and optimization of fermentation conditions for poly-gamma-glutamic acid production by *Bacillus subtilis* SCP017-03

**DOI:** 10.1371/journal.pone.0310556

**Published:** 2025-01-30

**Authors:** Caiyun Wu, Yutao Gou, Shuai Jing, Wei Li, Fanglan Ge, Jiao Li, Yao Ren

**Affiliations:** College of life Sciences, Sichuan Normal University, Chengdu, Sichuan, China; Universita degli Studi di Pavia, ITALY

## Abstract

Poly-gamma-glutamic acid (γ-PGA) is mainly synthesized by glutamate-dependent strains in the manufacturing industry. Therefore, understanding glutamate-dependent mechanisms is imperative. In this study, we first systematically analyzed the response of *Bacillus subtilis* SCP017-03 to glutamate addition by comparing transcriptomics and proteomics. The introduction of glutamate substantially altered gene expression within the central metabolic pathway of cellular carbon. Most genes in the pentose phosphate pathway (PPP), tricarboxylic acid (TCA) cycle, and energy-consuming phase of the glycolysis pathway (EMP) were down-regulated, whereas those in the energy-producing phase of glycolysis and those responsible for γ-PGA synthesis were up-regulated. Based on these findings, the fermentation conditions were optimized, and γ-PGA production was improved by incorporating oxygen carriers. In a batch-fed fermentor with glucose, the γ-PGA production reached 95.2 g/L, demonstrating its industrial production potential. This study not only elucidated the glutamate dependence mechanism of *Bacillus subtilis* but also identified a promising metabolic target for further enhancing γ-PGA production.

## Introduction

Poly-gamma-glutamic acid (γ-PGA) is a biopolymer naturally synthesized by microorganisms [1]. It possesses exceptional biochemical characteristics, including water absorption, moisture retention, and biocompatibility. As a novel, environmentally friendly biomaterial [2], it has extensive applications in various fields, including food, cosmetics, agriculture, medicine, and environmental fields [3, 4]. Therefore, γ-PGA biosynthesis has garnered considerable attention. γ-PGA-producing strains can be categorized into two groups: glutamate-dependent and glutamate-independent [5]. The former requires an external supply of glutamate to generate γ-PGA, and includes strains such as *Bacillus subtilis* NX-2 [5], *B*. *licheniformis* WX-02 [6], *B*. *subtilis* chungkookjang [7], and *B*. *subtilis* GXA-28 [8]. In contrast, glutamate-independent strains can produce γ-PGA without the addition of exogenous glutamate, synthesizing it de novo from carbon sources. Examples include *B*. *amyloliquefaciens* LL3 [9], *B*. *subtilis* C10 [10], and *B*. *licheniformis* GXG-5 [11]. Notably, glutamate-dependent strains exhibit higher γ-PGA production than glutamate-independent strains, making them the primary choice for industrial γ-PGA production [5].

The microbial γ-PGA synthesis pathway consists of three primary stages: precursor synthesis, polymerization transfer, and catabolism. In *B*. *subtilis*, key metabolic pathways, such as glycolysis (EMP), pentose phosphate pathway (PPP), tricarboxylic acid (TCA) cycle, and amino acid metabolism, play essential roles in γ-PGA biosynthesis [12]. L- glutamate, as a precursor, can be converted into D-glutamate through multiple enzymatic routes [13], as identified in *B*. *subtilis* IFO 3336 [14]. These L-glutamate and D-glutamate molecules undergo polymerization facilitated by the polymerization, a process that requires ATP consumption. The polyglutamic acid synthase (Pgs) is encoded by the *pgs* operon and encompasses four genes: *pgsB*, *pgsC*, *pgsA*, and *pgsE* in *B*. *licheniformis* and *B*. *amyloliquefaciens* [13, 15], with homologs in *B*. *anthracis* (*capB*, *C*, *A*, and *E*), and *B*. *subtilis* (*ywsC*, *ywtABC*) [16–18]. In addition, the gene encoding γ-PGA hydrolase (PgdS) is located downstream of the *pgs* operon in the *B*. *subtilis* (natto) genome [13]. Specifically, PgsB is an ATP-dependent amino ligase that catalyzes γ-PGA formation [18, 19]. PgsC is a cell membrane component of the γ-PGA synthesis system, featuring a structural resemblance to the N-acetyltransferase domain of N-acetylglutamic acid synthase [20]. PgsA contains a cell membrane-anchoring region [2] that may have implications for γ-PGA synthesis or transport [21, 22].

The regulatory mechanisms governing γ-PGA biosynthesis remain incompletely understood, with limited research in this area. Zeng et al. compared the genomic differences between glutamate-dependent and glutamate-independent strains, and identified 13 genes related to γ-PGA biosynthesis that were mutated in glutamate-dependent strains. However, the relationship between these mutations and glutamate dependence has not yet been conclusively established [11]. Sha et al. explored the dependence mechanism of *B*. *subtilis* NX-2 on glutamic acid during γ-PGA production through transcriptome analysis. Their findings revealed that the addition of glutamate significantly up-regulated genes associated with glycolysis, PPP, TCA cycle, glutamic acid synthesis, and γ-PGA synthesis. Overexpression of these genes causes the accumulation of γ-PGA, demonstrating the pivotal role of intracellular glutamic acid synthesis in regulating γ-PGA production in glutamate-dependent strains [23]. Li et al. preliminarily investigated the co-production mechanism of γ-PGA and nattokinase using transcriptomic technology. Their study revealed the up-regulation of genes related to carbohydrate metabolism. Furthermore, by analyzing major metabolic pathways, such as carbohydrate metabolism, potential target genes for enhancing γ-PGA production were identified [24].

Previous studies have shown that γ-PGA biosynthesis is regulated by two intracellular signal transduction mechanisms. ComA is a DNA-binding transcription factor and a corresponding regulator of the two-component ComP-ComA system. At high cell densities, the cell density signal of ComX is transferred from phosphorylated ComP to phosphorylated ComA. This event induces the expression of DegQ, which transfers the cell density signal to the DegS-DegU two-component system. This can promote DegU phosphorylation, and the combination of phosphorylated DegU with the pgsBCA gene promoter activates its expression [25, 26]. Phosphorylated DegU directly engages the *pgsBCA* promoter, thereby activating gene expression. Consequently, knockout of DegU and SwrA results in the loss of γ-PGA synthesis capability [27, 28]. SwrA and DegU facilitate the expression of the *pgsBCA* operon through protein-protein interactions, serving as key factors in activating its expression. However, the relationship between these regulatory factors and the addition of glutamate remains unclear [13, 23, 29, 30].

To elucidate the regulatory mechanisms governing γ-PGA biosynthesis, this study focused on the glutamate-dependent strain *B*. *subtilis* SCP017-03. The effects of exogenous glutamate supplementation on γ-PGA production and cell growth were investigated. Moreover, a comparison and analysis of the differential expression of bacteria at the transcription and protein levels in the presence and absence of glutamate was conducted using transcriptomics and proteomics technologies. This analysis focused on the differential expression observed in glycolysis, tricarboxylic acid cycle, pentose phosphate pathway, glutamic acid metabolism, and γ-PGA synthesis pathway, resulting from the addition of glutamate. Based on these findings, fermentation conditions were optimized, with a significant improvement in γ-PGA production. This study provides a comprehensive and systematic understanding of the molecular mechanisms underlying glutamate dependence in various γ-PGA strains. Furthermore, this study offers a valuable theoretical foundation for future gene modification endeavors to achieve high-yield γ-PGA production.

## Materials and methods

### Strain and media

*Bacillus subtilis* SCP017-03 (CGMCC 60083) was isolated and preserved in our laboratory. A single colony of SCP017-03 was cultivated in Luria-Bertani (LB) liquid medium and grown for 14–16 h at 37°C to produce a seed culture. The fermentation medium contained (g/L) glucose 60.0 g/L, yeast extract 12.0 g/L, sodium glutamate 50.0 g/L, KH_2_PO_4_ 0.5 g/L, K_2_HPO_4_ 0.5 g/L, and MgSO_4_ 0.1 g/L, with an initial pH 7.0. When used as a control for glutamate-dependent analysis, sodium glutamate was removed from the basic fermentation medium. In addition, glucose and yeast extract were replaced with other carbon and nitrogen sources, respectively when necessary. The seed culture (4%, v/v) was transferred into 50 mL of fermentation medium in 500 mL shaking flasks. Fermentation was performed at 37°C with agitation at 220 rpm for 72 h.

### Determination of related indexes in fermentation process

Cell growth was determined by measuring the optical density (OD) at 600 nm. In brief, the fermentation broth was diluted tenfold with sterilized distilled water (ddH_2_O), and the light absorption value at 600 nm was measured using a spectrophotometer (UV-8000), with sterile growth media at the same dilution used as a blank. The glutamate content in the fermentation broth was quantified using an enzyme-based method employing a biosensor (SBA-40c, Shan-Dong Academy of Sciences, China). The total sugar content in the fermentation broth was determined by diluting the broth to appropriate concentrations and using the sulfuric acid-acetone method [30]. The purification and concentration determination of γ-PGA were performed according to the method proposed by Chang et al. [31]. Briefly, 5 mL of fermentation broth was adjusted to pH 3 with 50% trichloroacetic acid and centrifuged at 10,000×g for 10 min to eliminate bacterial cells, and the pH of the supernatant was adjusted to 7. Four volumes of cold ethanol were added to the supernatant, and the mixture was stored overnight at 4°C. The precipitate was collected by centrifugation at 10000×g, resulting in crude γ-PGA that was further obtained by freeze-drying. The crude γ-PGA product was then dissolved in 5 mL of ddH_2_O, and its absorbance at 400 nm was measured using the hexadecyltrimethylammonium bromide (CTAB) method [31]. Finally, the molecular weight of γ-PGA was determined by sodium dodecyl sulfate—polyacrylamide gel electrophoresis (SDS-PAGE) [32].

### Transcriptomic analysis

Fresh seed cultures of strain SCP017-03 were inoculated into two types of fermentation media: one without glutamate and the other with glutamate, with each treatment conducted in three biological replicates. Cultures were maintained at 37°C with agitation at 220 rpm. After 20 h of fermentation, bacterial cells were collected by centrifugation and sent to Beijing Nuohe Zhiyuan Technology Co., Ltd. for Qualcomm RNA sequencing (RNA-Seq). A cDNA library was successfully constructed and sequenced using an Illumina NovaSeq 6000 high-throughput sequencing platform. Sequence data were deposited in the National Center for Biotechnology Information (NCBI) Sequence Read Archive (SRA, https://www.ncbi.nlm.nih.gov/sra) under the accession number PRJNA1057989. Quality control and data filtering were performed using FastQC software (Version 0.11.5). Low-quality RNA-Seq reads (reads with Qpred ≤ 20, representing more than 50% of the total reads) were removed. The filtered reads were subjected to genome mapping analysis using Bowtie 2 software [33] against the *B*. *subtilis* subsp. natto Best 195 for genome mapping analysis. Transcript assembly and quantification were performed using featureCounts in subread software. The relative quantitative value of transcripts was measured using fragments per kilobase of transcript per million mapped reads (FPKM), with transcripts having an FPKM value ≥ 1 considered effectively expressed. DESeq R package was used to identify differentially expressed genes (DEGs), with an absolute value of log2|Fold Change| ≥ 1 and a P _value_ < 0.05 as thresholds for determining significant differences in gene expression [34]. The sequences of all DEGs were aligned with sequences in the Gene Ontology (GO) and Kyoto Encyclopedia of Genes and Genomes (KEGG) databases to perform cluster and enrichment analyses using the Blastx tool (*E* value cut-off 10^−3^) [35]. An adjusted *p*< 0.05 was regarded as statistically significant.

### Proteomic analysis

The fermentation broth collected at the 6th and 20th hours was centrifuged and sent to Beijing Nuohe Zhiyuan Technology Co., Ltd. for further processing (Each treatment consisted of one biological replicate). Data are available via ProteomeXchange with the identifier PXD049242. The process included protein quality assessment, enzyme digestion, desalting, isobaric tags for relative and absolute quantification (iTRAQ) labeling, chromatographic separation, and mass spectrometry analysis. The mass spectrometry data were matched against the *B*. *subtilis168* protein database from NCBI, and the retrieval results were filtered using MaxQuant software. This filtering process selected spectral peptides with a reliability exceeding 99%, while peptides and proteins with false discovery rate (FDR) exceeding 0.01 were eliminated. Proteins showing up-regulated expression were identified when FC > 2.0, and P_value_ < 0.05. Conversely, down-regulated proteins were identified when FC < 0.50 and P_value_ < 0.05. Proteins were classified based on GO annotation and the KEGG database. Functional enrichment analysis of the differentially expressed proteins (DEPs) was performed using a two-tailed Fisher’s exact test, and GO terms and KEGG pathway with a corrected *p*-value *<* 0.05 were considered significant. All substrates obtained after enrichment were collated along with their *P* values, and then filtered for categories that were enriched in at least one of the clusters with a *P-*value < 0.01. This filtered P value matrix was transformed using the function x = −log10 (*P*-value). Finally, the x-values were z-transformed for each functional category. These z scores were then clustered by one-way hierarchical clustering (Euclidean distance and average linkage clustering) in Genesis. Cluster membership was visualized by a heat map using the “heatmap.2” function from the “gplots” R package.

### Quantitative real-time PCR assay

To validate the transcript sequence and analyze the temporal expression, genes involved in γ-PGA synthesis were mined from the *B*. *subtilis* SCP017-03 transcriptome database. Primer sequences for the target genes were designed using Primer Premier 5.0, with a total of 25 pairs (listed in S1 Table). RNA was extracted from all samples using a Quick RNA Isolation Kit (Huayueyang Biotechnology, Beijing, China). mRNA was reverse transcribed into complementary DNA (cDNA) following the instructions of the Superscript III First-Strand Synthesis System (Invitrogen, Carlsbad, USA). qRT-PCR analysis was conducted on a CFX Connect Real-Time PCR system (Bio-Rad, USA) by the ChamQ Universal SYBR qPCR Master Mix (Vazyme, China). The thermal cycling program was as follows: activation at 95°C for 3 min, followed by 40 cycles at 95°C for 20 s, 60°C for 20 s, and 72°C for 20 s. Each sample was run in triplicate. The 16S rRNA was used as an endogenous control, and quantitative real-time PCR data were analyzed using the comparative 2^-ΔΔCT^ method to quantify relative gene expression.

### Fed-batch cultures in a 5 L stirred fermentor

Fed-batch fermentation was performed in a 5-L fermentor (East Biotech, Zhenjiang, China) containing 2.5 L of medium. The optimized medium comprised 80 g/L glucose, 45 g/L L-glutamate, 0.5g/L K_2_HPO_4_, and 0.1 g/L MgSO_4_. Cultivation was conducted at 37°C with an airflow of 2.0 vvm. The dissolved oxygen level was maintained at 10% by adjusting the agitation rate from 250 to 500 rpm. NH_4_OH was automatically added to maintain a pH of 7.0. A glucose solution (200 g/L) was added to the fermentor at a flow rate of 8 mL/h to keep the sugar concentration no less than 10 g/L. Samples were collected periodically to determine cell growth (measured by OD_600_), sugar utilization, and products formation.

## Results

### Effects of glutamate addition on cell growth and γ-PGA synthesis

**Fig 1:**
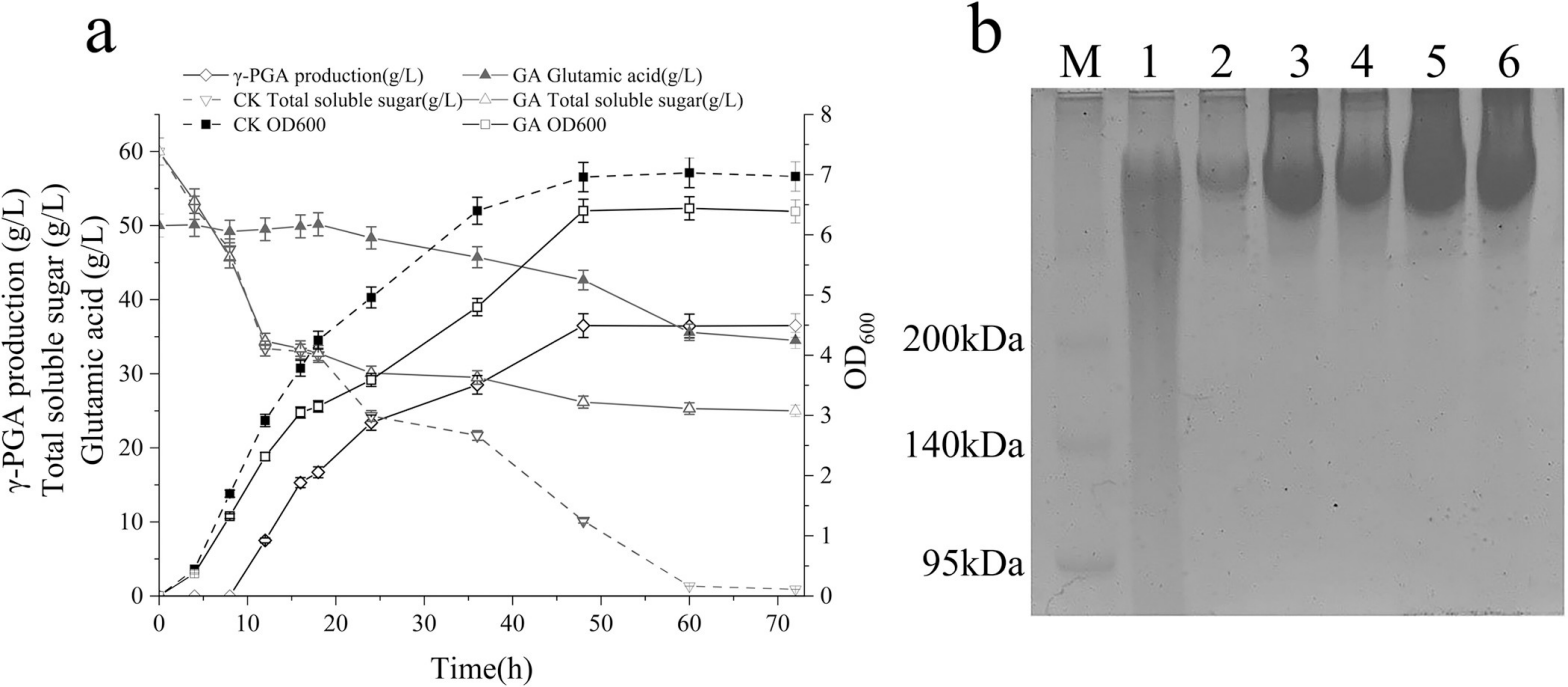
**Changes in glucose and glutamate concentrations, cell biomass, and accumulation of γ-PGA in strain SCP017-03 grown with glutamate at different fermentation times** (a), whereas γ-PGA was not produced without glutamate (data not shown). **SDS-PAGE analysis of γ-PGA** (b), where M is a molecular weight protein marker, and lanes 1–6 are the γ- PGA standard and γ-PGA samples after 20, 36, 48h, 60, and 72 of fermentation, respectively.

To assess the effect of glutamate addition on fermentation, *B*. *subtilis* SCP017-03 was cultured under two conditions: with glutamate addition (experimental group: GA) and without glutamate addition (control group: CK). Differences in glucose and glutamate consumption, cell growth, and γ-PGA production were compared. As depicted in Fig 1A, there were no significant differences in cell growth or carbon source consumption between the two groups during the initial 0–4 h of fermentation. However, after 10 h, the cell growth in the GA group lagged significantly behind that in the CK group. Between 18 and 48 h of fermentation, the differences in cell growth and glucose consumption between the experimental and control groups were more pronounced. At the 36-hour point, the OD_600_ value difference between the two groups reached a maximum, with CK in the control group at 6.4, while GA in the experimental group measured 4.8. After 12 h, glucose consumption in the GA group slowed, with the glucose content stabilizing at 25 g/L after 72-hour fermentation. However, the control group exhibited faster sugar consumption and exhausted glucose supply after 60th hour of fermentation. Sugar consumption correlated precisely with differences in cell growth. Especially, the CK group without adding glutamate did not synthesize γ-PGA during the fermentation, while γ-PGA synthesis was initiated in the GA group after 8 h of fermentation and continued to accumulate. After 18 h, the γ-PGA content reached 16.7 g/L, peaking at 36.5 g/L after 48 h of fermentation, remaining relatively stable until the end of fermentation. The residual concentration of glutamate in the fermentation broth of the GA group gradually decreased from 50 to 35 g/L after 60 h of fermentation, indicating a modest reduction of 15 g/L over the course of fermentation. This observation suggested that the bacterial cells of this strain synthesized a certain amount of glutamate.

γ-PGA generated in the fermentation broth was assessed using SDS-PAGE (Fig 1B). The figure illustrates that the molecular weight of γ-PGA produced by this strain significantly exceeded 200 kDa. However, as fermentation progressed, there was a slight reduction in the molecular weight.

### Transcriptome analysis of strain *scp017-03* in response to glutamate addition

RNA samples from each group were sequenced using the Illumina NovaSeq 6000 high-throughput sequencing platform. The sequencing output data exhibited base quality values of Q30 and Q20 exceeding 95% and 98%, respectively, with a sample sequencing error rate of less than 0.03%. These metrics complied with stringent quality standards for Illumina sequencing. Table 1 shows the alignment results of each sample against the reference genome. All six samples achieved a total mapping rate, were successfully aligned to the genome via sequence mapping, and each exhibited unique alignment positions on the reference sequence.

**Table 1:** Mapping results of strain SCP017-0 transcriptome.

Sample.name	CK1	CK2	CK3	GA1	GA2	GA3
Total reads	8754356	7523564	7800772	7724204	7734902	7438034
Total mapped reads	8621421	7418727	7684771	7636601	7638796	7325040
Uniquely mapped reads	8425126	7275289	7526589	7500556	7495239	7168957
Multiple mapped reads	196295	143438	158182	136045	143557	156083
Total mapping rate	98.48%	98.61%	98.51%	98.87%	98.76%	98.48%
Uniquely mapping rate	96.24%	96.7%	96.49%	97.1%	96.9%	96.38%
Multiple mapping rate	2.24%	1.91%	2.03%	1.76%	1.86%	2.1%

**Fig 2:**
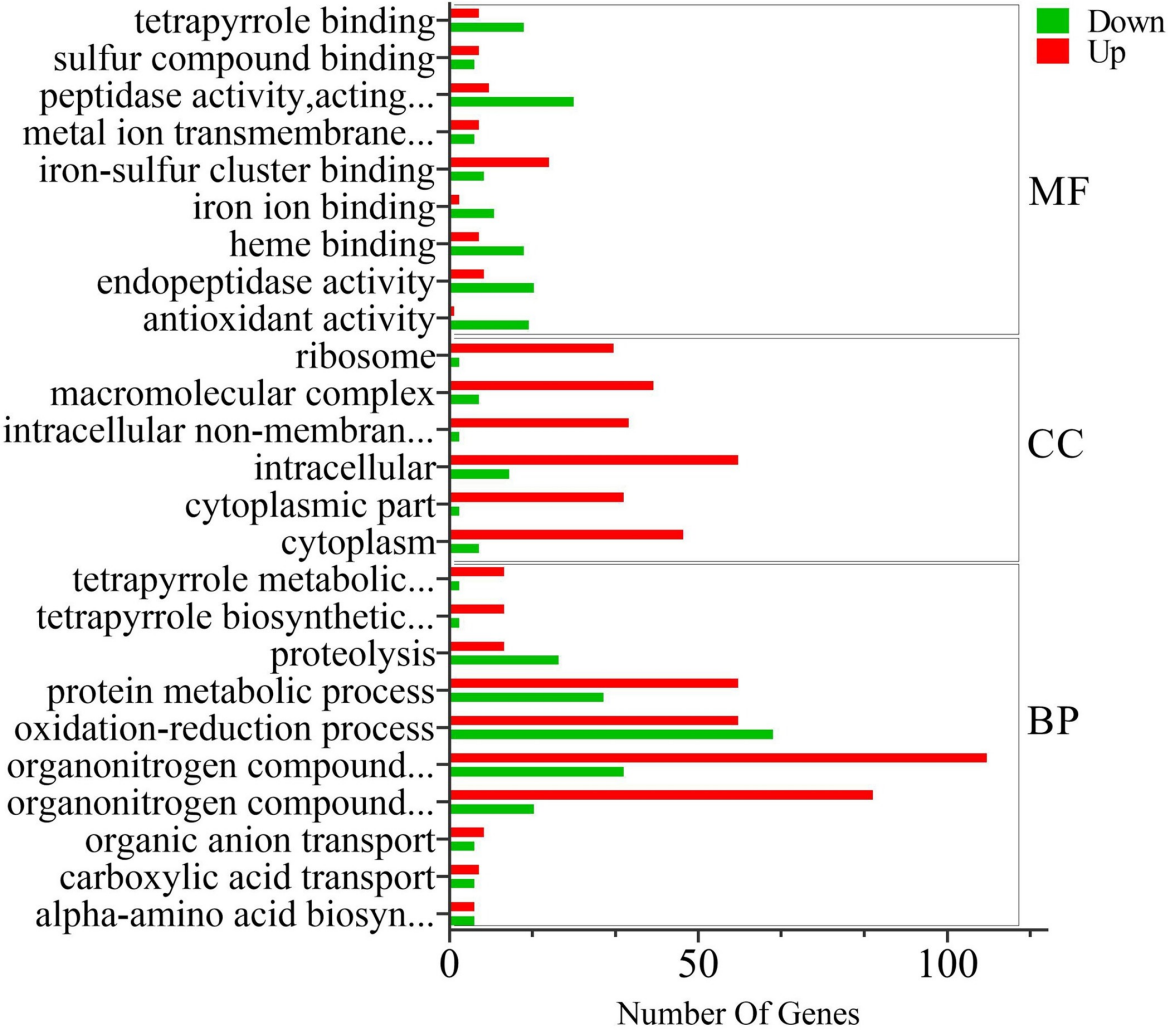
Bar chart illustrating GO enrichment analysis of differentially expressed genes. The horizontal axis represents the number of up-regulated and down-regulated genes in the GO Term, and the vertical axis represents the GO Term.

**Fig 3:**
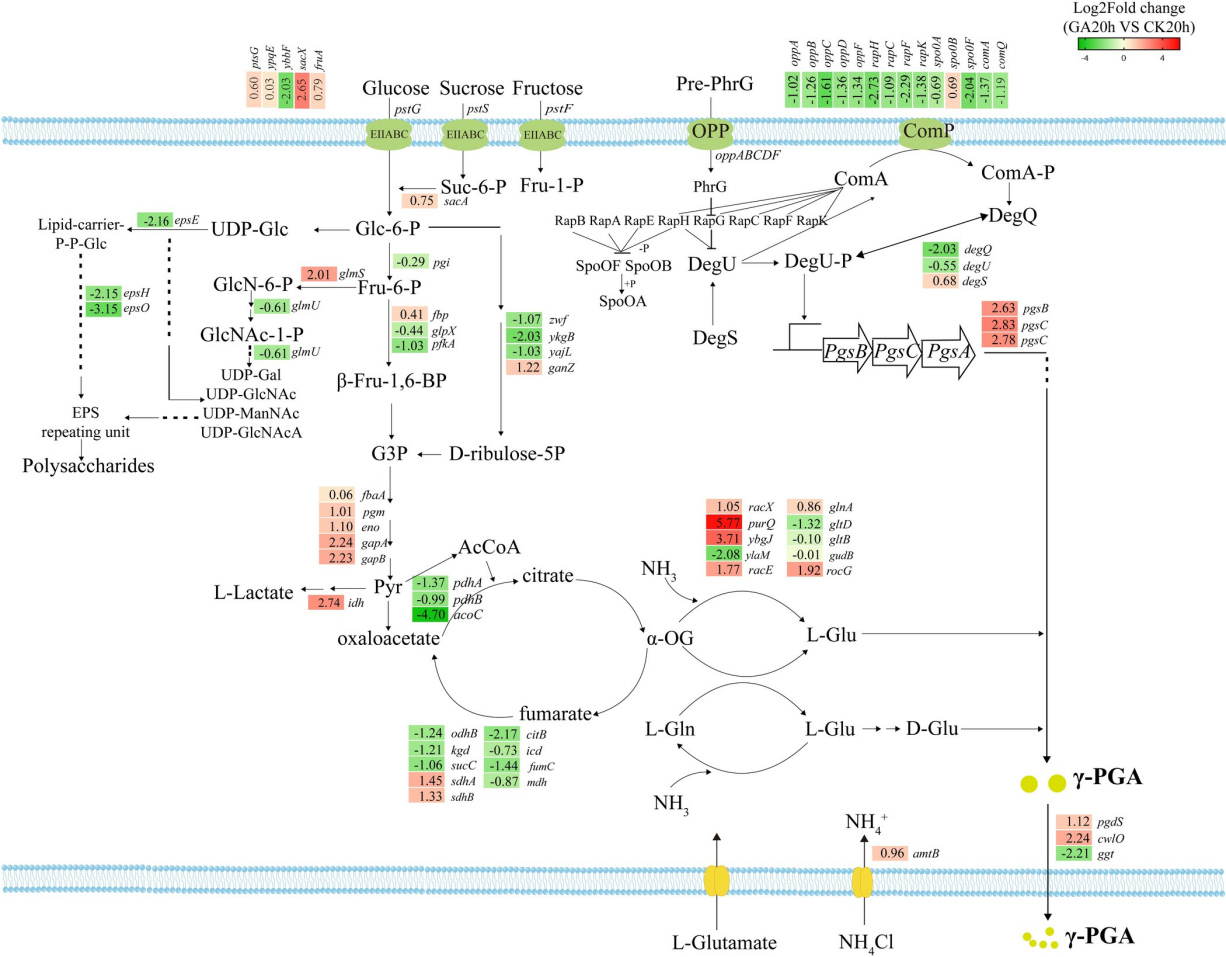
Transcription profiles of key genes involved in glycolysis, PPP, TCA cycle, glutamate synthesis, and γ-PGA synthesis in strain SCP017-03 grown with and without glutamate. Numbers represent expression ratios (log_2_FC). Red indicates up-regulation, and green indicates down-regulation. Definitions: *ptsG*, *ypqE*, *ybbF*, *sacX*, and *fruA* encode phosphotransferase system; *sacA* encodes sucrose-6-phosphate hydrolase; *epsE*, *epsH*, and *epsO* encode glycosyltransferase; *glmS* encodes glutamine-fructose-6-phosphate transaminase; *glmU* encodes glucosamine-1-phosphate N-acetyltransferase; *pgi* encodes glucose-6-phosphate isomerase; *fbp* encodes Fur-regulated basic protein FbpA; *glpX* encodes bacterial fructose-1,6-bisphosphatase; *pfkA* encodes 6-phosphofructokinase; *zwf* encodes glucose-6-phosphate dehydrogenase; *ykgB* encodes 6-phosphogluconolactonase; *yajL* encodes NADP-dependent phosphogluconate dehydrogenase; *ganZ* encodes decarboxylating NADP(+)-dependent phosphogluconate dehydrogenase; *fbaA* encodes fructose-bisphosphate aldolase; *pgm* encodes phosphoglyceromutase; *eno* encodes phosphopyruvate hydratase; *gapA* and *gapB* encode glyceraldehyde-3-phosphate dehydrogenase; *idh* encodes L-lactate dehydrogenase; *pdhA* encodes E1α subunit of pyruvate dehydrogenase; *pdhB* encodes E1β subunit of pyruvate dehydrogenase; *acoC* and *odhB* encodes 2-oxoacid dehydrogenases acyltransferase; *kgd* encodes alpha-ketoglutarate decarboxylase; *sucC* encodes succinyl-CoA synthetase subunit beta; *sdhA* encodes succinate dehydrogenase; *citB* encodes aconitate hydratase; *icd* encodes isocitrate dehydrogenase; *fumC* encodes fumarate hydratase; *mdh* encodes malate dehydrogenase; *racX* encodes broad specificity amino-acid racemase RacX; *purQ* encodes phosphoribosylformylglycinamidine synthase subunit PurQ; *ybgJ* encodes glutaminas; *ylaM* encodes glutaminase A; *racE* encodes glutamate racemase; *glnA* encodes type I glutamate—ammonia ligase; *gltD* encodes glutamate synthase small subunit; *gltB* encodes glutamate synthase large subunit; *gudB* encodes cryptic glutamate dehydrogenase (GDH); *rocG* encodes glutamate dehydrogenase (GDH); *DegQ* encodes pleiotropic regulator; *DegU* encodes two-component response regulator; *DegS* encodes two-component sensor histidine kinase; *pgsB*, *pgsC*, and *pgsA* encode γ-PGA synthetase operon; *comA* encodes two-component system response regulator ComA; *comP* encodes two-component system response regulator ComP; *oppABCDF* encode oligopeptide ABC transporter substrate-binding protein; *rapHCFK* encode response regulator aspartate phosphatase; *spo0ABF* transcription factor Spo0A; and *spo0BF* encode sporulation initiation phosphotransferase Spo0F.

A total of 1867 differentially expressed genes were identified, comprising 862 up-regulated and 1005 down-regulated genes. GO functional enrichment analysis indicated the presence of both up-regulated and down-regulated genes within three categories: biological process (BP), cellular component (CC), and molecular function (MF) (Fig 2). These findings suggest that the addition of exogenous glutamate significantly affected various metabolic pathways in this strain. Furthermore, the enrichment results of KEGG pathway analysis encompassed the differentially expressed genes and certain genes related to γ-PGA synthesis (Fig 3).

The phosphotransferase system (PTS) is the primary mechanism employed by bacteria to uptake hexose, hexitol, disaccharides, and other carbohydrates. As shown in Fig 3, the addition of exogenous glutamate led to the differential expression of *ybbF* and *sacX* genes within the sucrose metabolism system (EIIBCA or EIIBC component). Their log_2_FC values were -2.03 and +2.65, respectively. The carbohydrates transported into the membrane entered not only the central metabolic pathway but also polysaccharide synthesis (EPS). Within the EMP pathway consisting of energy consumption and productivity stages, gene expression in the energy consumption stage, including *pgi* (glucose-6-phosphate isomerase), *glpX* (fructose 1,6-diphosphatase), and *pfkA* (ATP-dependent phosphofructokinase), was slightly down-regulated. Conversely, during the production stage of Pyr synthesis from G-3-P, the expression of these five genes was significantly up-regulated. These genes included *fbaA* (fructose-diphosphate aldolase), *pgm* (glycerophosphate mutase), *eno* (enolase), *gapA*, and *gapB* (glyceraldehyde-3-phosphate dehydrogenase). Consequently, the addition of exogenous glutamate enhanced the EMP pathway. In the PPP pathway, the expression of *zwf* (glucose-6-phosphate dehydrogenase) and *ykgB* (glucose-6-phosphate esterase) was down-regulated with a log_2_FC (fold change) of -1.07 and -2.03, respectively, whereas *ganZ* (6-glucose-6-phosphate dehydrogenase) witnessed an up-regulation in expression (Fig 3).

The TCA cycle is initiated by condensation of acetyl-CoA and oxaloacetic acid to produce citric acid. Acetyl-CoA is formed through the oxidative decarboxylation of pyruvate under aerobic conditions. As shown in Fig 3, most genes in the TCA cycle pathway were down-regulated in the GA group. Notably, the log_2_FC values for *citZ* and *icd* were -1.76 and -0.72, respectively, with only the *sdhABC* gene (encoding succinate dehydrogenase) displaying up-regulation. In addition, the coding genes responsible for the pyruvate dehydrogenase complex, which catalyzes the decarboxylation of pyruvate to synthesize acetyl coenzyme A, were down-regulated. For example, the *acoC* gene (encoding dihydrooctylamide transacetylase) exhibited down-regulation with a log_2_FC (fold change) of -4.69, while the *idh* gene (encoding lactate dehydrogenase), responsible for transforming pyruvate into lactic acid, was up-regulated with a log_2_FC (fold change) of 2.74 (Fig 3). These findings suggested that the synthesis of high-viscosity γ-PGA by cells resulted in reduced dissolved oxygen levels in the culture solution, thereby reducing metabolic flux through the tricarboxylic acid pathway.

L-glutamic acid is converted into D-glutamic acid through the catalysis of RacE (glutamate racemase), and these compounds are subsequently utilized in the synthesis of γ-PGA by γ-PGA synthetase. Fig 3 demonstrates an up-regulation in the expression of the *glnA* gene (encoding glutamine synthetase) and *racE*, with log_2_FC values of 0.86 and 1.77, respectively. This upregulation enhanced the production of the precursor D-glutamic acid for γ-PGA, thereby strengthening γ-PGA synthesis. Notably, the *pgsBCA* gene cluster (encoding γ-PGA synthetase) was significantly up-regulated, with log_2_FC values of 2.63, 2.83, and 2.78, respectively. The *pgsBCA* operon is regulated by DegS-DegU and ComA-ComP two-component systems. In the CK group, γ-PGA production was not observed without glutamate (data not not shown). The FPKM value of the *degU* gene was 2396, and the FPKM values of the *pgsBCA* gene cluster were 859, 744, and 1243, indicating the baseline expression of the γ-PGA synthesis system in the absence of exogenous glutamate. However, the *pgsBCA* operon was further activated upon the addition of exogenous glutamate, with the FPKM value of the *pgsA* gene reaching 8533. These two-component systems can interact with phosphorylated ComA to induce degQ expression. Nevertheless, the expression levels of *comA*, *comX*, *comQ*, and *degQ* genes in this strain remained low. For example, the FPKM value of *degQ* in the GA group was only 69. After γ-PGA synthesis, the cells required the action of γ-PGA hydrolase to release γ-PGA. This process may involve three genes, *pgdS*, *cwlO*, and *ggt*. Transcriptome analysis revealed that these three genes exhibited low expression in the GA group, with FPKM values of 27, 282, and 266, respectively. Furthermore, the expression of *ggt* in the CK group was significantly higher than that in the GA group.

### qPCR analysis of genes related to γ-PGA biosynthesis

Selected genes related to γ-PGA biosynthesis were subjected to qPCR analysis to validate the findings of transcriptomic analysis. To investigate dynamic changes in key genes and regulatory factors during fermentation, samples were collected for qPCR analysis at three time points: 10 h (immediately following γ-PGA synthesis initiation), 20 h, and 48 h (when γ-PGA accumulation peaked). The results are presented in Table 2. After 10-hour fermentation, most genes in the TCA cycle, PPP pathway, and glutamic acid synthesis pathway exhibited slight down-regulation, except for a few genes such as *citA*, *gltP*, and *rocA*. In the γ-PGA synthesis module, only the expression of the regulator DegQ showed a slight decrease (log_2_FC: -0.67), and genes encoding γ-PGA synthetase were significantly up-regulated. Specifically, *pgsB* expression increased by 4.94 times, and *pgsA* expression increased by 4.63 times. Moreover, the genes *pgdS* and *cwlO*, associated with γ-PGA hydrolysis, were up-regulated, with fold changes of 2.73 and 1.47, respectively. At the 20-hour point, genes in the central metabolic pathway generally showed down-regulation, except for the *eno* gene in the EMP pathway and the *sdhA* gene in the TCA cycle, consistent with the transcriptome analysis results. Notably, *pgdS* exhibited slight down-regulation. In addition, the γ-PGA synthesis module consistently displayed up-regulated expression. In the late stage of fermentation (48 h), owing to significant nutrient depletion in the culture medium, gene expression in the central pathway decreased. Concurrently, as γ-PGA accumulation peaked, some γ-PGA was hydrolyzed by the bacterial cells to maintain its state, as evidenced by the substantial upregulation of *cwlO* gene expression, with a fold change of 21.93. Throughout the fermentation process, the regulatory factor degQ was consistently down-regulated, whereas *degS* and *degU* were up-regulated, indicating complex interactions within the DegS-DegU two-component system.

**Table 2:** Comparison of expression of key genes in γ-PGA synthesis by qPCR of strain SCP017-03 grown with glutamate and without glutamate at different fermentation times.

Gene	10h	20h	48h
*pfkA*	4.30	0.60	2.18
*pgi*	2.93	0.75	1.63
*eno*	6.30	2.97	2.18
*pdhA*	2.52	0.05	0.11
*citB*	1.87	1.99	0.54
*icd*	1.50	0.40	0.53
*fumC*	2.69	0.44	0.43
*ykgB*	1.06	0.23	0.22
*zwf*	1.97	0.88	3.34
*ganZ*	2.80	0.47	0.20
*gltP*	0.67	0.16	0.04
*rocA*	0.20	2.86	5.96
*racE*	4.42	0.35	0.01
*degQ*	0.67	0.32	0.05
*degU*	1.36	1.02	1.41
*degS*	7.59	2.03	1.13
*pgsA*	4.63	2.45	18.83
*pgsB*	4.94	4.82	21.26
*pgsC*	3.23	9.38	12.21
*glnA*	0.92	1.64	0.94
*putM*	1.55	0.03	0.06
*pgdS*	2.73	0.76	1.71
*comA*	2.45	0.58	6.70
*cwlO*	1.47	4.89	21.93

Notes: The numbers represent the value of relative expression level (2^-ΔΔCT^) of genes in SCP017-03 strain grown with glutamate comparing without glutamate (P < 0.01).

### Proteomics analysis of strain SCP017-03 in response to glutamate addition

**Fig 4:**
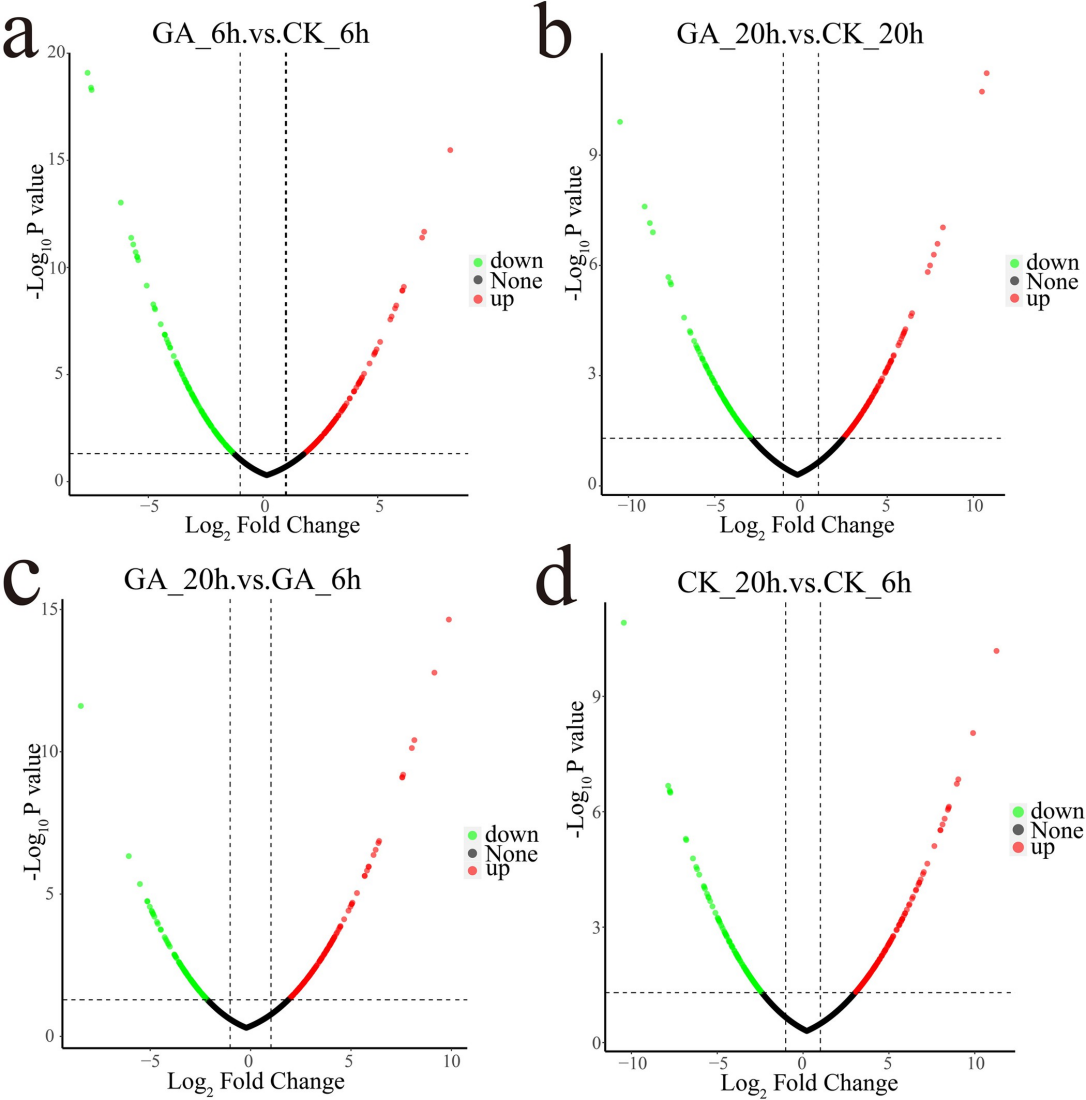
Volcano plots depicting differential protein expression. A: Comparison of protein levels between strains with and without glutamate at the 6th hour of fermentation. B: Comparison of protein levels between strains with and without glutamate at the 20th hour. C: Comparison of protein levels between strains at the 20th and 6th hours of fermentation with glutamate. D: Comparison of protein levels between strains at the 20th and 6th hours of fermentation without glutamate. The horizontal axis represents the multiple differences (log_2_ P_value_) of differentially expressed proteins, and the vertical axis represents the P_value_ (-log_10_ P_value_). Black represents proteins with non-significant differences, red represents significantly up-regulated proteins, and green represents significantly down-regulated proteins.

Strain SCP017-03 was subjected to fermentation in culture medium with or without exogenous glutamate (GA and CK groups). Cell samples were collected at 6 and 20 h after fermentation. Protein analysis was conducted using 4D-label-free technology, leading to the identification of 2,161 proteins and 13,243 peptides. Each sample (CK6h, GA6h, CK20h, and GA20h) was then quantified. After 6 h of fermentation, comparison between the GA and CK groups (Fig 4A) revealed 328 differentially expressed proteins. These included 162 up-regulated and 166 down-regulated proteins. After 20 h of fermentation (Fig 4B), 279 differentially expressed proteins were identified, comprising 142 up-regulated and 137 down-regulated proteins. A comparison within the GA group, specifically between 20 and 6 h of fermentation (Fig 4C), yielded 297 differentially expressed proteins. Among them, 158 were up-regulated and 139 were down-regulated. Similarly, 296 differentially expressed proteins were observed in the CK group (Fig 4D), including 162 up-regulated and 136 down-regulated proteins. The volcano plot of the differential proteins visually illustrated that the majority of the proteins (represented in black) exhibited no significant differences. Proteins with differences in expression are denoted in red (up-regulated) and green(down-regulated) (Fig 4).

**Fig 5:**
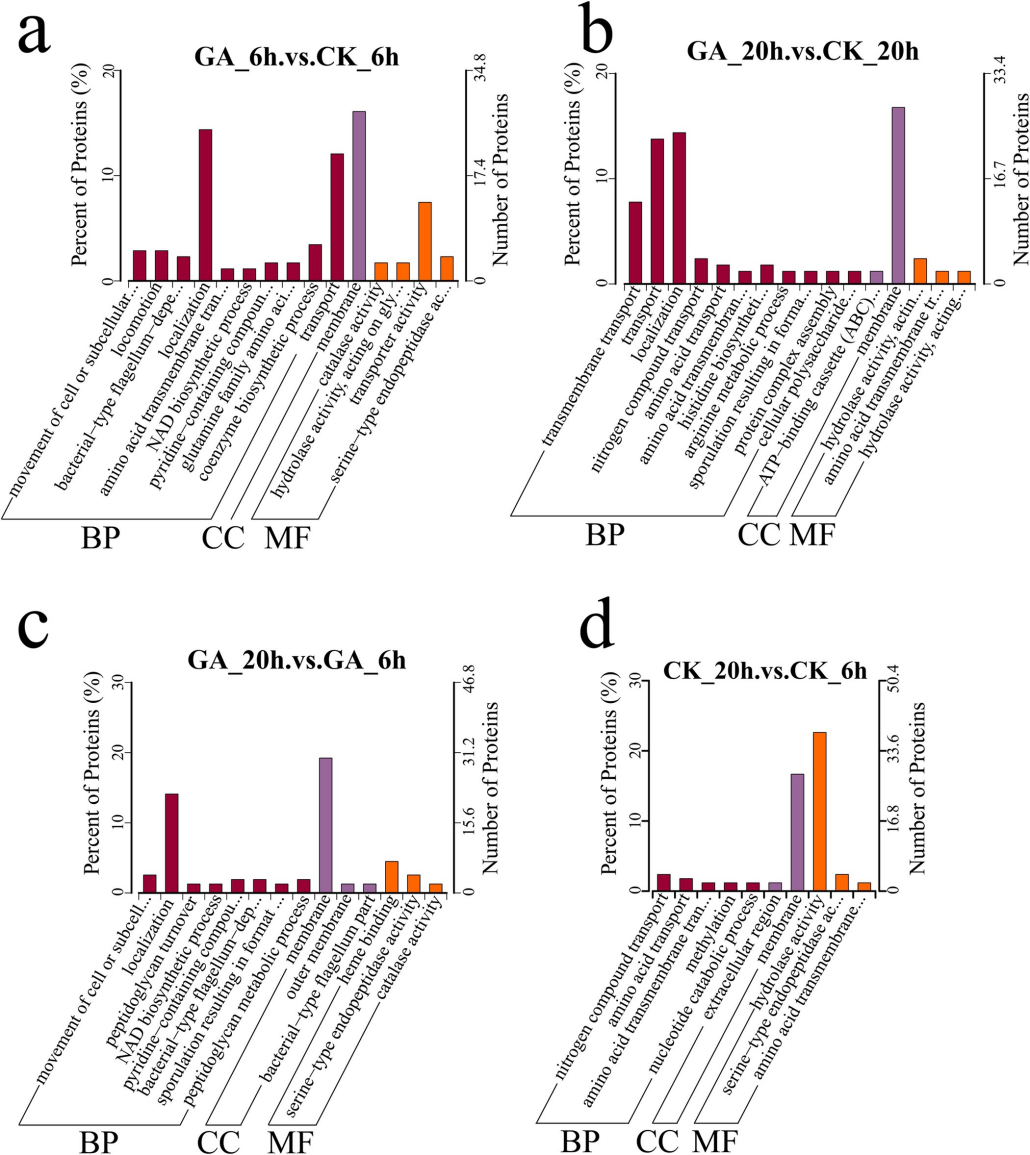
Statistical diagram of GO secondary annotation classification for differentially expressed proteins. A: Differential protein GO enrichment of strains with and without glutamate at the 6th hour of fermentation. B: Differential protein GO enrichment of strains with and without glutamate at the 20th hour of fermentation. C: Differential protein GO enrichment of the strain at the 20th and 6th hours of fermentation with glutamate. D: Differential protein GO enrichment at the 20th and 6th hours of fermentation without glutamate. The horizontal axis represents GO entries enriched with differential proteins, and the vertical axis represents the number of differentially expressed proteins annotated to a certain GO entry.

The enrichment category of differential proteins GO in the GA group exhibited significant changes between the 6-hour and 20-hour fermentation periods, whereas the corresponding changes in the CK group were relatively minor (Fig 5A and 5B). Compared to the differentially expressed proteins associated with BP in the CK group, those in the GA group at 6 h of fermentation were primarily involved in processes related to localization (25 proteins), transportation (21 proteins), and coenzyme biosynthesis (6 proteins). Furthermore, differentially expressed proteins linked to “cellular component (CC)” were predominantly associated with specific cellular structures. As the fermentation duration reached 20 h, the BP processes in the GA group emphasized transmembrane transport (13 proteins), transport (23 proteins), and localization (24 proteins), whereas the CC processes centered on membrane-related components (28 proteins) and the ABC transport complex (2 proteins). In the MF category, the focus shifted towards hydrolase activity (4 proteins). Similarly, the analysis of GO enrichment for differential proteins in both the GA and CK groups (Fig 5C and 5D) revealed that the differentially expressed proteins in the GA group were primarily concentrated in the BP (22 identified proteins) and CC (30 membrane-related proteins) categories, whereas in the CK group, the emphasis was mainly on the CC (28 membrane-related proteins) and MF (38 proteins associated with hydrolase activities) categories.

S2 Table illustrates that after 6 h of fermentation, protein expression within the central metabolic pathways of the GA group exhibited slight changes compared to the CK group. For example, in the EMP pathway, except for GlpX (fructose 1,6-diphosphatase), which showed a slight up-regulation (log_2_FC: 0.31), other proteins displayed down-regulated expression. Notably, Pgi, Fbp, and Eno had log_2_FC values of -0.58, -0.93, and -0.82, respectively. In the TCA cycle, only PdhB (β subunit of pyruvate dehydrogenase E1 component) and PDHC (α subunit of DNA polymerase III) exhibited slight increases in protein expression, with log_2_FC values of 0.47 and 0.59, respectively. Conversely, the expression of other proteins, including SdhA (protein subunit of succinate dehydrogenase), notably decreased, with a log_2_FC value of -1.25. Changes in protein expression within the PPP pathway were minimal. After 20-hour fermentation, the log_2_FC values for certain proteins in these metabolic pathways underwent substantial changes, with most proteins in the EMP pathway showing increased expression. In the TCA cycle, aside from PdhABC, which was up-regulated by 0.71, 1.38, and 1.16, the expression of other proteins aligned with the transcriptome results, indicating down-regulation. Similarly, the expression of PPP pathway proteins was consistent with the results of transcriptome analysis. Furthermore, this study analyzed protein expression at different stages of fermentation (20 and 6 h) for different treatments. The results revealed that in the GA group, protein expression in the EMP pathway was predominantly up-regulated, whereas the CK group exhibited the opposite trend. However, enzyme expression in the TCA cycle and PPP pathway remained largely consistent between the two groups, with PPP pathway enzymes being down-regulated.

After a 6-hour fermentation, comparison between the GA and CK groups revealed notable changes in the expression of proteins involved in the glutamate synthesis pathway (S2 Table). Specifically, the expressions of RacE and RocG in the glutamate synthesis pathway were up-regulated in the GA group, with log_2_FC values of 1.78 and 0.15, respectively. In contrast, the expression of other proteins within this pathway decreased, with GltB (glutamate synthase domain 3) showing the most significant down-regulation, marked by a log_2_FC value of -3.28. However, proteins and regulatory factors related to γ-PGA synthesis exhibited only slight changes, such as the log_2_FC values of -0.39 for DegQ, -0.78, DegU, and -0.19 for PgsA. However, after a 20-hour fermentation period, substantial changes were observed in the expression of histone-like proteins in both the GA and CK groups. In the glutamic acid synthesis pathway, the log_2_FC value of PurQ increased from -0.29 to 1.89, while RacE exhibited an increase in log_2_FC from 1.76 to 4.12. Concurrently, the expression of other proteins in this pathway was also down-regulated. Within the γ-PGA synthesis system, both PgsA and PgsB were up-regulated, with log_2_FC values of 1.68 and 3.05, respectively. Notably, the protein expression of DegQ and DegS in the DegS-DegU two-component system diverged from the transcriptome analysis. Specifically, the log_2_FC values for regulatory factors DegQ and DegS were 1.9 and -0.44, respectively. However, the protein expression of the ComP-ComA system aligned with the aforementioned transcriptional analysis, with the ComA regulatory factor exhibiting down-regulation. Additionally, genes involved in the glutamate synthesis pathway generally exhibited increased at protein levels. For example, the expression of glutamate racemase (RacE), responsible for the conversion of D-glutamic acid to L-glutamic acid, increased by 4.12 times. In contrast, the expression of γ-PGA hydrolase (Pgds) significantly decreased (log_2_FC: 2.24) (S2 Table).

Differential protein analysis indicated a significant increase in the activity of γ-PGA polymerase in the GA group after 20 h of fermentation compared to 6 h (S2 Table). Notably, the log_2_FC values for PgsA and PgsB were 2.04 and 3.83, respectively. Furthermore, it was observed that γ-PGA synthetase activity in the CK group after 20 h of fermentation was lower than that at the 6-hour mark. γ-PGA synthetase activity was notably increased by 2.05-fold. This observation suggested that the expression of γ-PGA synthetase genes remained relatively stable at the transcriptional level, even in the absence of exogenous glutamic acid. However, the translation of this mRNA into protein may have been influenced by other genes or regulatory factors, resulting in the reduced activity of γ-PGA synthetase.

### Optimization of fermentation conditions

**Fig 6:**
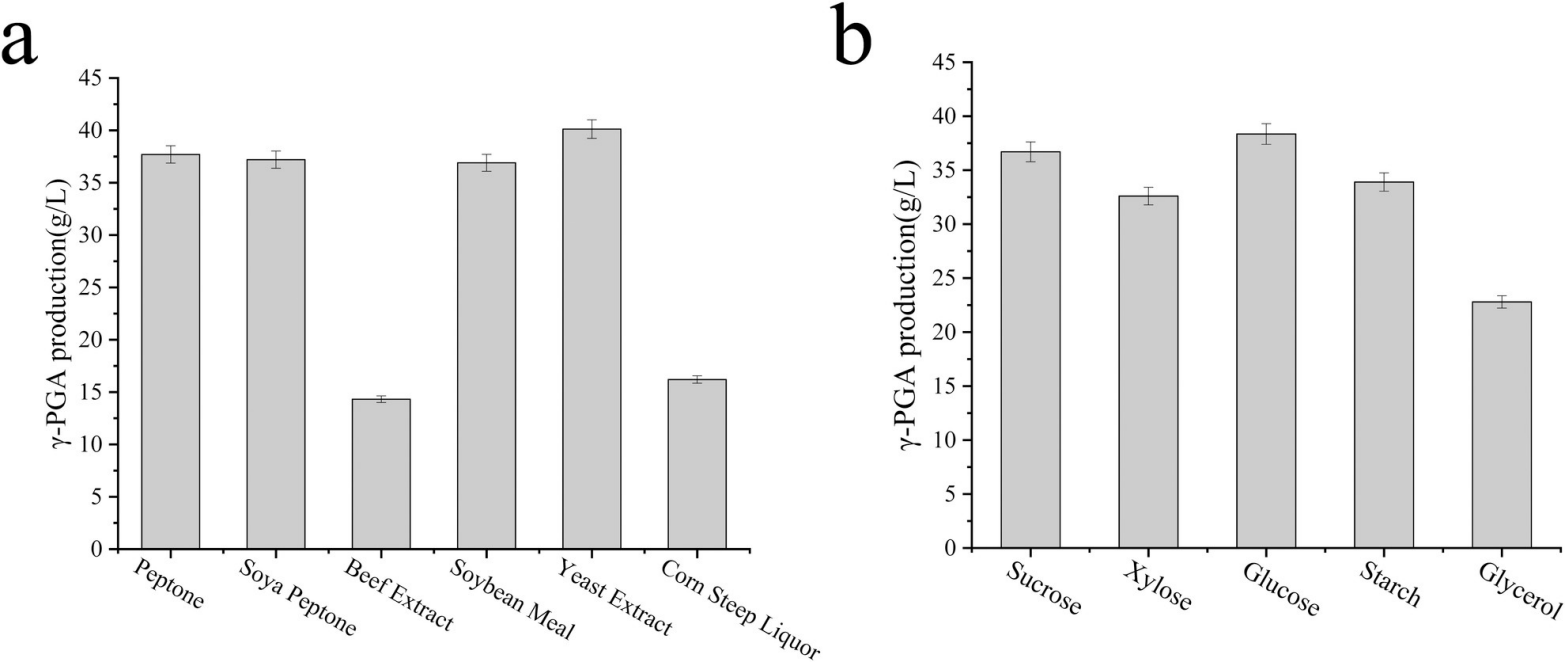
Effects of different carbon (A) and nitrogen (B) sources on γ-PGA production. Data represent the average of three replicates, and error bars correspond to standard deviations.

Preliminary optimization was conducted for carbon and nitrogen sources to enhance γ-PGA production during fermentation. The initial concentrations of the carbon and nitrogen sources were 60 g/L and 12 g/L, respectively. Fermentation was performed at 37°C with agitation at 220 rpm for 72 h, and γ-PGA production was measured. The results of carbon source optimization are shown in Fig 6A. Among the tested options, glucose as the carbon source yielded the highest γ-PGA production at 38.35 g/L, followed by sucrose (36.5 g/L), starch (33.9 g/L), and xylose (32.6 g/L). Conversely, when glycerol was employed as the carbon source, γ-PGA production was the lowest at 22.8 g/L. Fig 6B illustrates that yeast extract was a superior nitrogen source, yielding a γ-PGA production of 40.12 g/L. Other nitrogen sources, including pancreatic peptone, soybean peptone, and bean cake powder, yielded approximately 37.5 g/L of γ-PGA. In contrast, beef paste and corn steep liquor, when applied as nitrogen sources, resulted in lower productions of 14.32 g/L and 16.2 g/L, respectively. Consequently, the optimal carbon source was glucose, and the preferred nitrogen source was yeast extract.

**Fig 7:**
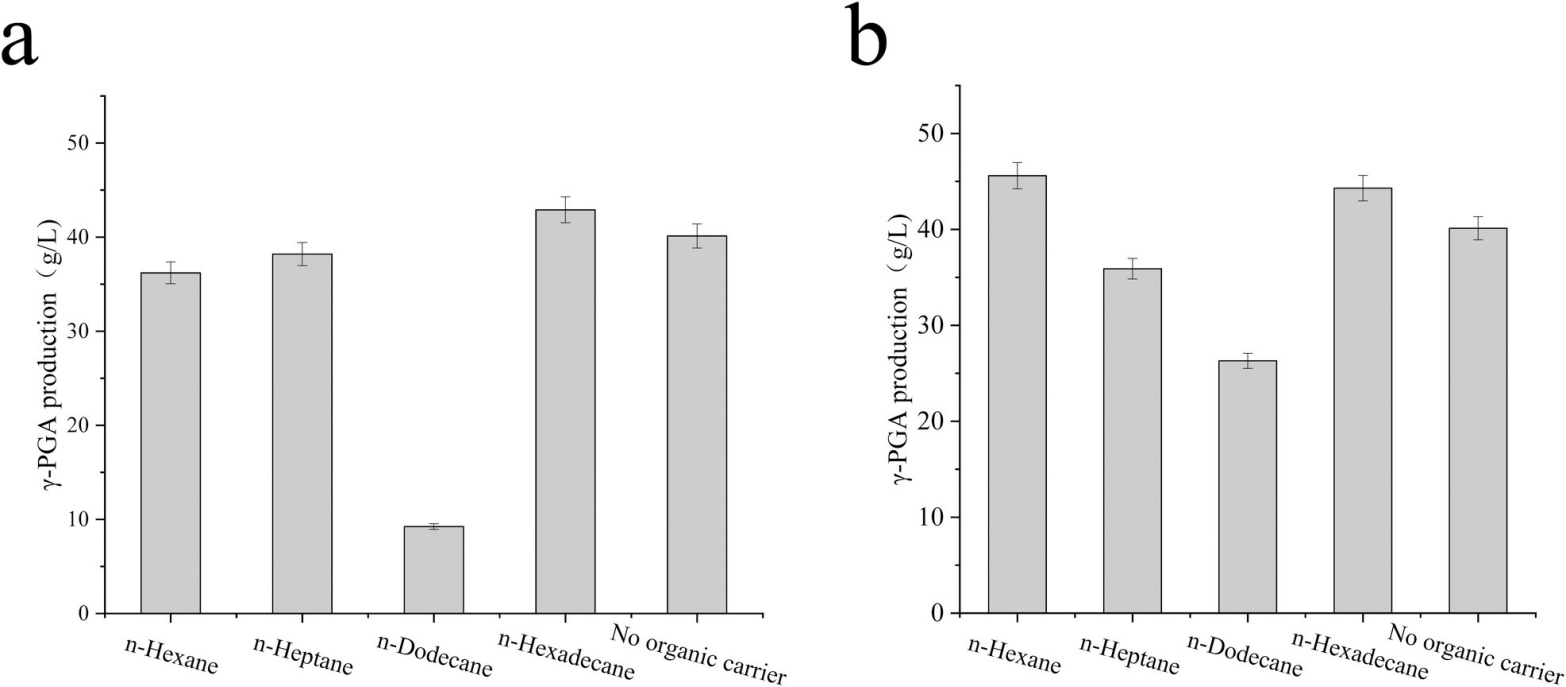
Effects of different organic oxygen carriers (a) and their addition time (b) on γ-PGA production. (a) Added at the beginning of fermentation and (b) Added after 24 h of fermentation.

The results revealed that during the synthesis of γ-PGA, the transcriptional activity of related genes, including those associated with glycolysis, tricarboxylic acid cycle, and PPP pathway, decreased. This decrease could be related to the increased fermentation broth viscosity and the impact of dissolved oxygen. To optimize the fermentation, four organic carriers (w/v, 0.5%) were selected. The organic carriers included n-hexane, n-heptane, n-dodecane, and n-hexadecane. Fig 7a illustrates that when n-hexadecane was introduced at the onset of fermentation, the highest γ-PGA production was achieved, reaching 42.9 g/L. This was followed by n- heptane and n- hexane, with γ-PGA yields of 38.2 g/L and 36.2 g/L, respectively. In contrast, the group with the addition of n-dodecane showed the lowest γ-PGA production (9.24 g/L). Considering the potential impact on bacterial growth, an organic carrier was not added to the fermentation broth until the 24th hour of fermentation. Results showed that compared with groups where the organic carrier was added at the initial stage of fermentation, γ-PGA production increased by 9.4 g/L in the n-hexane group and by 17.06 g/L in the n-dodecane group (Fig 7A and 7B). Among these, the n-heptane group achieved the highest γ-PGA output of 45.6 g/L. Notably, no significant difference was observed between the n-hexadecane and n-heptane groups, with respective outputs of 43.3 g/L and 35.9 g/L (Fig 7A and 7B). Therefore, n-hexane was selected for fermentation for 24 h.

### Fed-batch fermentation in a 5 L fermentor

**Fig 8:**
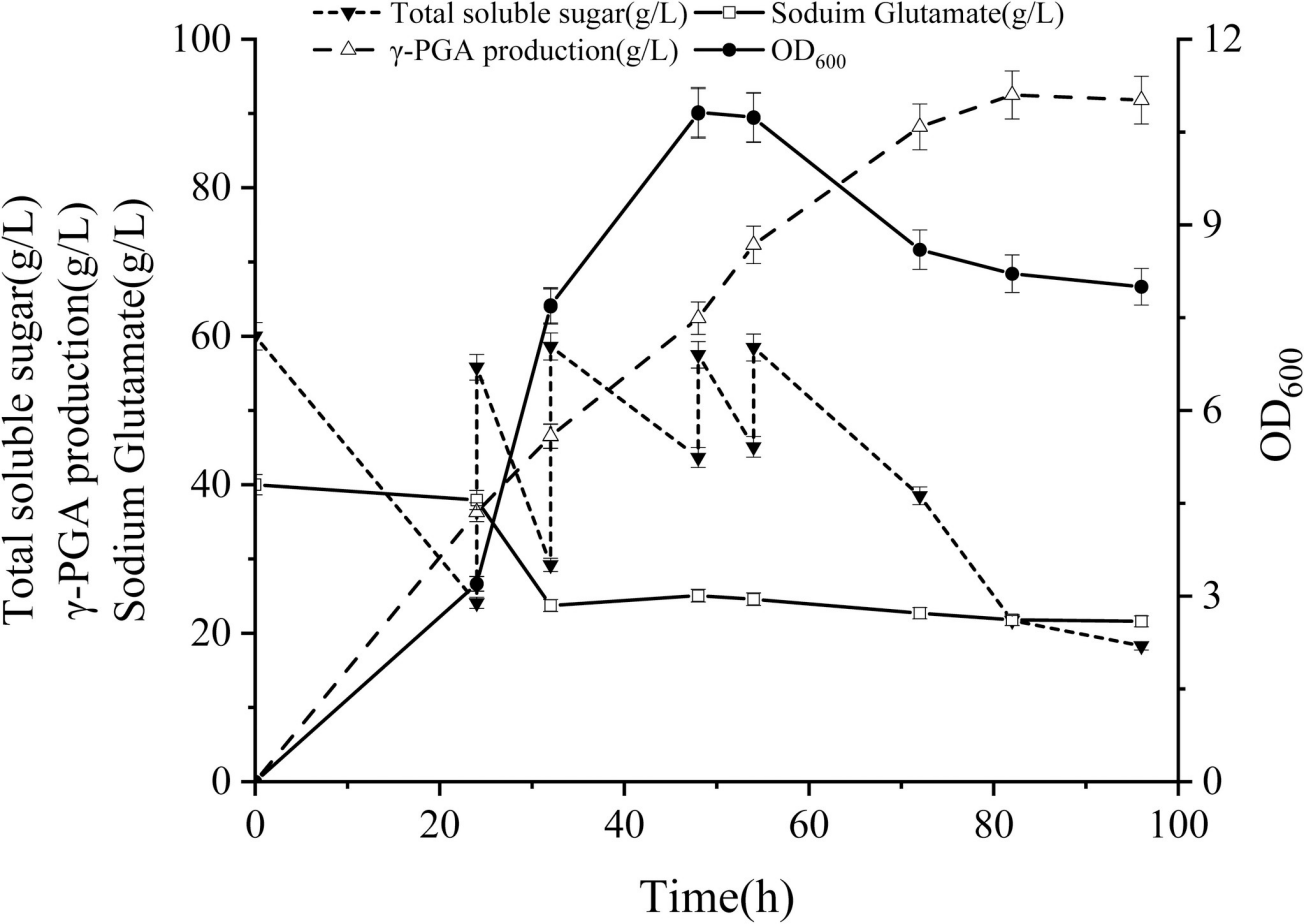
Changes in glucose and glutamate concentration and cell biomass during γ-PGA production in 5 L fed-batch fermentation. Error bars indicate the standard deviation of three biological replicates.

In the context of shaking flask conditions, the fermentation duration was insufficient, resulting in low dissolved oxygen concentration and inadequate carbon source concentration in the later stages. These limitations limit the successful synthesis of γ-PGA. Therefore, fermentation processes were optimized using a 5 L fermentor. The fermentation parameters were set to 500 rpm for agitation, 2 L/min for aeration, and pH of 7. Sucrose was added when the total sugar content decreased below 25 g/L. Fig 8 illustrates the dynamic changes in various fermentation parameters. After 24 h of fermentation, the cell growth (OD_600_) reached 3.195, total sugar residue was 24.09 g/L, and γ-PGA production reached 36.29 g/L. However, glutamate content in the fermentation broth remained relatively stable. At this point, an additional 100 g of glucose was added, increasing the total sugar content to 55.82 g/L. By the 32-hour point, the glutamate in the fermentation broth decreased to 33.72 g/L, indicating a consumption of 16.27 g/L. Bacterial cell growth increased to 7.68, prompting the second addition of 100 g of glucose. At the 48-hour point, bacterial growth (OD_600_) peaked at 10.82, whereas the total sugar residue reached 43.67 g/L. To sustain fermentation, 50 g of glucose was added. During the fermentation period from 54 to 96 h, as the bacterial cells entered the decay phase and nutrient consumption decreased, the last feeding occurred at 54 h, with 50 g of glucose added. After 82 h of fermentation, the total glucose consumption reached 84.18 g/L, and γ-PGA production peaked at 92.5 g/L, subsequently stabilizing. Towards the end of fermentation, bacterial growth (OD_600_) decreased to approximately 8.0, with total sugar and glutamate levels in the tank measuring 18.3 g/L and 31.6 g/L, respectively.

## Discussion

Currently, industrial production of γ-PGA primarily relies on glutamate-dependent strains, necessitating the addition of exogenous sodium glutamate for γ-PGA synthesis. Therefore, thorough investigation of the mechanisms underlying glutamate dependence is crucial. However, research in this area has been limited. In this study, the response of strain SCP017-03 to glutamate addition was investigated using transcriptomics and proteomics. In contrast to the findings of Sha et al. [23], which nearly all genes in the three central metabolic pathways were significantly up-regulated, our transcriptome results showed a significant down-regulation in the expression of coding genes in the PPP and TCA cycle pathways. Conversely, most coding genes in the EMP pathway were up-regulated. This disparity was attributed to decreased dissolved oxygen in the culture medium caused by the highly viscous γ-PGA product. This decreased the expression of genes encoding the TCA and PPP pathways. However, most coding genes in the glycolytic pathway were up-regulated, promoting pyruvate accumulation and directing metabolism towards the fermentation product, lactic acid. The significant up-regulation of dehydrogenase-encoding genes upon glutamic acid addition further validated this hypothesis. Building upon this analysis, subsequent experiments in this study enhanced γ-PGA production by increasing the dissolved oxygen levels in the culture medium. Interestingly, the *pgsBCA* gene cluster responsible for γ-PGA synthesis was present in the genome of the control group without exogenous glutamic acid, with FPKM values reaching 859, 744, and 1243, respectively, although γ-PGA synthesis was not observed. This suggested that the lack of sufficient glutamic acid as a precursor within the cells and protein-level regulation may be contributing factors. In contrast, the experimental group supplemented with exogenous glutamate exhibited significant up-regulation in the expression of the *pgsBCA* gene cluster, with log_2_FC values of 2.63, 2.83, and 2.78, respectively, which is consistent with the findings of Sha et al. [23]. In summary, the response mechanism to glutamate addition varied among different γ-PGA-producing strains, highlighting the complexity of this phenomenon.

The gene encoding the γ-PGA degrading enzyme *pgdS* is located downstream of the *pgsBACE* operon. PgdS is secreted into the extracellular space, and facilitates γ-PGA degradation. Additionally, previous studies have shown that γ-glutamyltranspeptidase (GGT) and DL-endopeptidase (CwlO) are also related to the degradation of γ-PGA [36–38]. In this study, observations were made in both the control group (CK), lacking glutamate, and the experimental group (GA), with glutamate supplementation. *pgdS* exhibited minimal expression in both groups, whereas the expression of *cwlO* remained low, with FPKM values of 59 and 282, respectively. However, the expression level of *ggt* decreased, with its FPKM dropping from 1231 to 266. Feng et al. conducted individual knockout experiments of the *pgdS*, *ggt*, and *cwlO* genes in glutamate-independent strains. γ-PGA production improved only when the *cwlO* gene was singly knocked out, and further enhancements were observed through simultaneous knockout of the *pgdS* and *ggt* genes [39]. Similarly, Scoffone et al. reported doubling of γ-PGA production after double knockout of *pgdS* and *ggt* in glutamate-dependent strains [40]. Furthermore, overexpression of *pgdS* efficiently biosynthesizes low-molecular-weight γ-PGA [41].

Proteomic analysis in this study revealed that genes in the energy consumption stage of the glycolytic pathway exhibited a slight down-regulation, whereas genes associated with the energy production stage were up-regulated. Furthermore, the PPP and TCA cycle pathways displayed noticeable down-regulation, aligned perfectly with the metabolic pathway responses observed in transcriptome sequencing following glutamate supplementation. Additionally, dynamic changes in the expression of key genes involved in γ-PGA synthesis were analyzed using qPCR at various fermentation time points. The results indicated that during the initial stage of fermentation (10 h), genes from all central metabolic and γ-PGA synthesis pathways were up-regulated. As fermentation progressed (20 h and 48 h), the genes associated with γ-PGA synthesis continued to display up-regulation, while those related to central metabolic pathways exhibited a declining trend. In summary, for glutamic acid-dependent bacteria, the addition of glutamate had a dual purpose: activating γ-PGA synthetase expression and providing the ample supply of glutamate necessary for γ-PGA synthesis.

The mechanism underlying the high yield of γ-PGA is complex and encompasses aspects such as glutamate supply, γ-PGA synthesis, transport, secretion, energy metabolism, and glutamate metabolism. In this study, qPCR analysis revealed that in the experimental group supplemented with glutamate, the expression of the *pgsBCA* gene cluster exhibited significant up-regulation throughout the 10th to 48th hours of fermentation, compared to the control group. Moreover, the genes *degS* and *degU* displayed substantial up-regulation at the 10th and 20th hours of fermentation, with a slight increase at the 48th hour, while *degQ* showed down-regulation. Transcriptome analysis indicated that the coding genes *comX*, *comA*, and *swrA* were expressed at low levels during the 20th hour of fermentation and were slightly down-regulated after glutamate addition. In parallel, proteomic analysis demonstrated a significant up-regulation of *comP* expression at the sixth hour of fermentation. Moreover, the quorum sensing system (QS system), which is known to influence γ-PGA synthesis by primarily affecting *comA* expression and indirectly regulating γ-PGA synthesis, has been considered [42, 43]. The results indicated significant down-regulation of the coding genes for Spo0K permease (*oppABCDF*) and phosphatase RapC (*rapHCFK*), whereas the expression of *comA* was slightly down-regulated. This suggested that *comA* may be regulated by other regulatory factors or genes. In the 5-L fermentor, 50 g/L of sodium glutamate was initially added to the culture medium, and 31.6 g/L remained at the end of fermentation. Meanwhile, the γ-PGA output reached 92 g/L, indicating that the glutamate supplied for γ-PGA synthesis primarily originated from de novo synthesis through carbon catabolism, rather than from exogenous glutamate supplementation. This differs from the findings of Sha et al. [23], in which γ-PGA was derived mainly from exogenous glutamate. Therefore, the main role of exogenous glutamate appears be to stimulate the expression of genes related to γ-PGA synthesis in the strain SCP017-03. Additionally, several regulatory factors including DegU, DegQ, DegS, ComP-ComA, and SwrA exhibited differential expression.

Based on a comprehensive analysis of strain SCP017-03 at the physiological, protein, and transcription levels, along with the optimization of fermentation medium composition and enhanced dissolved oxygen conditions, several key findings emerged. Glucose and yeast extract were identified as the optimal carbon and nitrogen sources, respectively, which increased the production of γ-PGA from 36.5 g/L to 40.12 g/L. However, the accumulation of high viscosity γ-PGA in fermentation broth may hinder oxygen transfer, thereby negatively affecting cell growth and γ-PGA production [5]. Various strategies have been proposed to address this issue. For instance, Su et al. integrated the *vgb* gene (encoding hemoglobin) into the genome of a γ-PGA-producing strain [44] to augment dissolved oxygen levels. Zhang et al. successfully increased γ-PGA production to 39.4 g/L by introducing four different organic oxygen carriers into the fermentation medium [5]. Feng et al. proposed an aerobic plant cellulose experimental bed to enhance γ-PGA production [45]. In this study, we focused on promptly mitigating dissolved oxygen deficiency in fermentation cultures and enhancing γ-PGA production by optimizing oxygen carriers. The results demonstrated a significant increase in γ-PGA production by introducing n-heptane during a 24-hour fermentation period, increasing the production from 40.12 g/L to 44.3 g/L. Furthermore, in a 5 L fermentor, the production reached an impressive 95.2 g/L after batch feeding with glucose.

In summary, this study presented a pioneering integration of transcriptome and proteome analyses, revealing the glutamate-dependent mechanism underlying γ-PGA synthesis in *B*. *subtilis*. Through a comparative analysis of strains cultured with and without glutamate, key genes involved in glycolysis, PPP, TCA cycle, and γ-PGA synthesis pathway were identified. This exploration revealed the potential factors and metabolic pathways crucial for γ-PGA production, thereby providing an initial framework for understanding the production mechanism of γ-PGA. Subsequent research focused on fermentation optimization, resulting in a remarkable output of 95.2 g/L with the introduction of an oxygen carrier, demonstrating promising prospects for industrial-scale production. The findings of this study offer a valuable metabolic target for further enhancement of γ-PGA production, with substantial significance for large-scale production of targeted metabolites.

## Supporting information

S1 Table Primer sequences used in qPCR experiments. (DOCX)

S2 Table Protein expression levels of various metabolic pathways at different fermentation times. (DOCX)

S1 Raw image (PDF)
