# Supplementary material for: Analysis of glutamate-dependent mechanism and optimization of fermentation conditions for poly-gamma-glutamic acid production by Bacillus subtilis SCP017-03
Source: PLoS One. 2025 Jan 30;20(1):e0310556. doi: 10.1371/journal.pone.0310556 (PMC11781620; doi:10.1371/journal.pone.0310556)
Supplement: S1 Table — (DOCX) [file pone.0310556.s001.docx]

| Table S1 Primer sequences used in qPCR experiments | | | | |
| --- | --- | --- | --- | --- |
| Primers | Primer sequence (5' to 3') | | | |
| *pfkA*-F1 | GCGCAGTAGTCAGAAAAGCG | | | |
| *pfkA*-R1 | GATCAATCCCGCGTATCCGT | | | |
| *pgi*-F1 | CAACTGATAAAGAGCGCGGC | | | |
| *pgi*-R1 | CGCCGACATCGTCAGGAATA | | | |
| *eno*-F1 | ATGAACATCGTAAACGGCGG | | | |
| *eno*-R1 | TCGTCACCTACAGCAGTGTT | | | |
| *pdhA*-F1 | CTCCTACTGCGGGTCAAGAA | | | |
| *pdhA*-R1 | AGGCCGTGCCAAATTAACTG | | | |
| *citB*-F1 | AAAGATACGCCTGCCGGAAA | | | |
| *citB*-R1 | GTTACTTCACCAGTCGGCCA | | | |
| *icd*-F1 | CGAAGGAGACGGAACTGGTC | | | |
| *icd*-R1 | GGGAGCCACTCACCTGTTTT | | | |
| *fumC*-F1 | GCCGCTGTTTTGGCTGTTTA | | | |
| *fumC*-R1 | TCCTGAAGATGTGTGCGTCC | | | |
| *ykgB*-F1 | GAAGGAAACTGGCCTCGTGA | | | |
| *ykgB*-R1 | CCGGGTAAGGGACAGAAACC | | | |
| *zwf*-F1 | CAGATGGGCTGGTGTTCCAT | | | |
| *zwf*-R1 | TTTGATTGGCTGTGCGTGTG | | | |
| *gntZ*-F1 | TGCAGGCGATTGATAACGGT | | | |
| *gntZ*-R1 | TTGAGCTGCCATCCGTTCTT | | | |
| *gltP*-F1 | GGCATTGAACTCCTCCTCCC | | | |
| *gltP*-R1 | GAGACCGACAAGCGGAAAGA | | | |
| *rocA*-F1 | ACCGTGGAAGGAAGCAGATG | | | |
| *rocA*-R1 | CGCGAGCGGGAAGTTAAATG | | | |
| *racE*-F1 | TGAACCAGTCTCCGATTGCC | | | |
| *racE*-R1 | ACATTCCACATGCCCGACTT | | | |
| *degQ*-F1 | TGGAAACGATGGAAAAGAAACTTG |  |  |  |
| *degQ*-R1 | TGTTTCGTAATGAATCTGTCGTTTC |  |  |  |
| *degU*-F1 | CAAGCGGAGTTTCTGCACAC | | | |
| *degU*-R1 | TGCTTTTTCCGTCTGCAAGC | | | |
| *degS*-F1 | GATGATCCAGCAGCGTGAGA | | | |
| *degS*-R1 | CTCTTCTTCCTGCGCCTCAA | | | |
| *pgsA*-F1 | TGCGGACAAACTGCTGGTAT | | | |
| *pgsA*-R1 | GTTCCGGCTAAGACAAGCCT | | | |
| *ywsC*-F1 | CGCGGAAAATCGACTGTGAC | | | |
| *ywsC*-R1 | AATCGCGTTAGCCCCTCTTT | | | |
| *ywtA*-F1 | TCGTGCCAGGTTTAATTGCC | | | |
| *ywtA*-R1 | AAAGGTCGCTCCGCTCAATA | | | |
| *glnA*-F1 | GAGCAAAGAAGAGCGCATGG | | | |
| *glnA*-R1 | GCGCTTTGACCATGACTTCG | | | |
| *yusM*-F1 | TGCAGTTGTTAAGCGGGAAC | | | |
| *yusM*-R1 | ACGCATTCTGTAGCCTTCCT | | | |
| *ywtD*-F1 | CCTTACAAACAGGGCGGAGT | | | |
| *ywtD*-R1 | TTCCGGCATTGTACTGGCTT | | | |
| *comA*-F1 | GCTGTCATGGAAGGCACCAA | | | |
| *comA*-R1 | CTCGCCGCCTAGATTCAGAT | | | |
| *cwlO*-F1 | ACTTGGTTTGGCTTCCGTCA | | | |
| *cwlO*-R1 | TCCGCCGATGCAGTTTTACT | | | |
| *16s RNA*-F1 | ACGGTCGCAAGACTGAAACT | | | |
| *16s RNA*-R1 | CCTGGTAAGGTTCTTCGCGT | | | |
