# Supplementary material for: Analysis of glutamate-dependent mechanism and optimization of fermentation conditions for poly-gamma-glutamic acid production by Bacillus subtilis SCP017-03
Source: PLoS One. 2025 Jan 30;20(1):e0310556. doi: 10.1371/journal.pone.0310556 (PMC11781620; doi:10.1371/journal.pone.0310556)
Supplement: S2 Table — (DOCX) [file pone.0310556.s002.docx]

| Table S2 Protein expression levels of various metabolic pathways at different fermentation times. | | | | |
| --- | --- | --- | --- | --- |
| Protein | GA6h VS CK6h | GA20h VS CK20h | GA20h VS GA6h | CK20h VS CK6h |
| Pgi | -0.58 | 0.3 | 0.38 | -0.5 |
| Fbp | -0.93 | 1.54 | 0.95 | -1.52 |
| PfkA | -0.42 | 0.4 | -0.23 | -1.05 |
| FbaA | -0.82 | 0.97 | 0.48 | -1.31 |
| GlpX | 0.31 | -0.2 | -0.44 | 0.06 |
| Eno | -0.82 | 0.96 | 0.48 | -1.31 |
| GapA | -0.28 | 0.7 | 0.63 | -0.35 |
| PdhA | -0.06 | 0.71 | -0.94 | -1.71 |
| PdhB | 0.47 | 1.38 | -0.37 | -1.28 |
| PdhC | 0.59 | 1.16 | -1.01 | -1.65 |
| CitZ | -0.81 | -1.57 | 1.46 | 2.21 |
| CitB | -1.08 | -1.77 | 0.08 | 0.77 |
| Icd | -0.62 | -0.7 | 0.71 | 0.79 |
| OdhB | -0.07 | -1.36 | -0.6 | 0.68 |
| SucC | 0.02 | -1.23 | 0.15 | 1.42 |
| SucD | 0.09 | -1.9 | 0.01 | 2.01 |
| SdhA | -1.25 | 1.32 | 1.89 | 0.77 |
| SdhB | 0.2 | 0.52 | 0.74 | 0.79 |
| SdhC | -0.81 | -0.42 | 1.23 | 0.68 |
| fumC | 0.14 | -0.37 | -0.37 | 0.14 |
| Mdh | -0.18 | -0.13 | 0.5 | 0.45 |
| Zwf | 0.05 | 0.12 | -0.71 | -0.78 |
| GanZ | -0.05 | 0.7 | -0.54 | -1.29 |
| GltD | -2.11 | -1.62 | 2.13 | 1.64 |
| RocG | 0.15 | - | - | 1.03 |
| GltB | -3.28 | -1.85 | 2.45 | 1.02 |
| PutM | -0.67 | -4.05 | 0.33 | 2.87 |
| PurQ | -0.29 | 1.89 | -0.87 | -3.05 |
| CwlO | -1.06 | 2.24 | 2.34 | -0.95 |
| RacE | 1.76 | 4.12 | -1.63 | -4 |
| PgsA | -0.19 | 1.68 | 2.04 | -0.01 |
| PgdS | 0.7 | -2.24 | -0.89 | 2.05 |
| PgsB | -0.29 | 3.05 | 3.83 | 0.48 |
| DegQ | -0.39 | 1.9 | 0.64 | -1.65 |
| DegS | -0.04 | -0.44 | 0.64 | 1.04 |
| DegU | -0.78 | -0.62 | 1.47 | 1.31 |
| ComP | 0.79 | 1.81 | 0.4 | -0.62 |
| ComA | 0.04 | -0.92 | -0.76 | 0.2 |
| SwrA | -1.18 | -0.45 | 1.81 | 1.07 |

Notes: the numbers indicate the value of log_2_fold change.
