# Supplementary figures and images for: Analysis of glutamate-dependent mechanism and optimization of fermentation conditions for poly-gamma-glutamic acid production by Bacillus subtilis SCP017-03
Source: PLoS One. 2025 Jan 30;20(1):e0310556. doi: 10.1371/journal.pone.0310556 (PMC11781620; doi:10.1371/journal.pone.0310556)

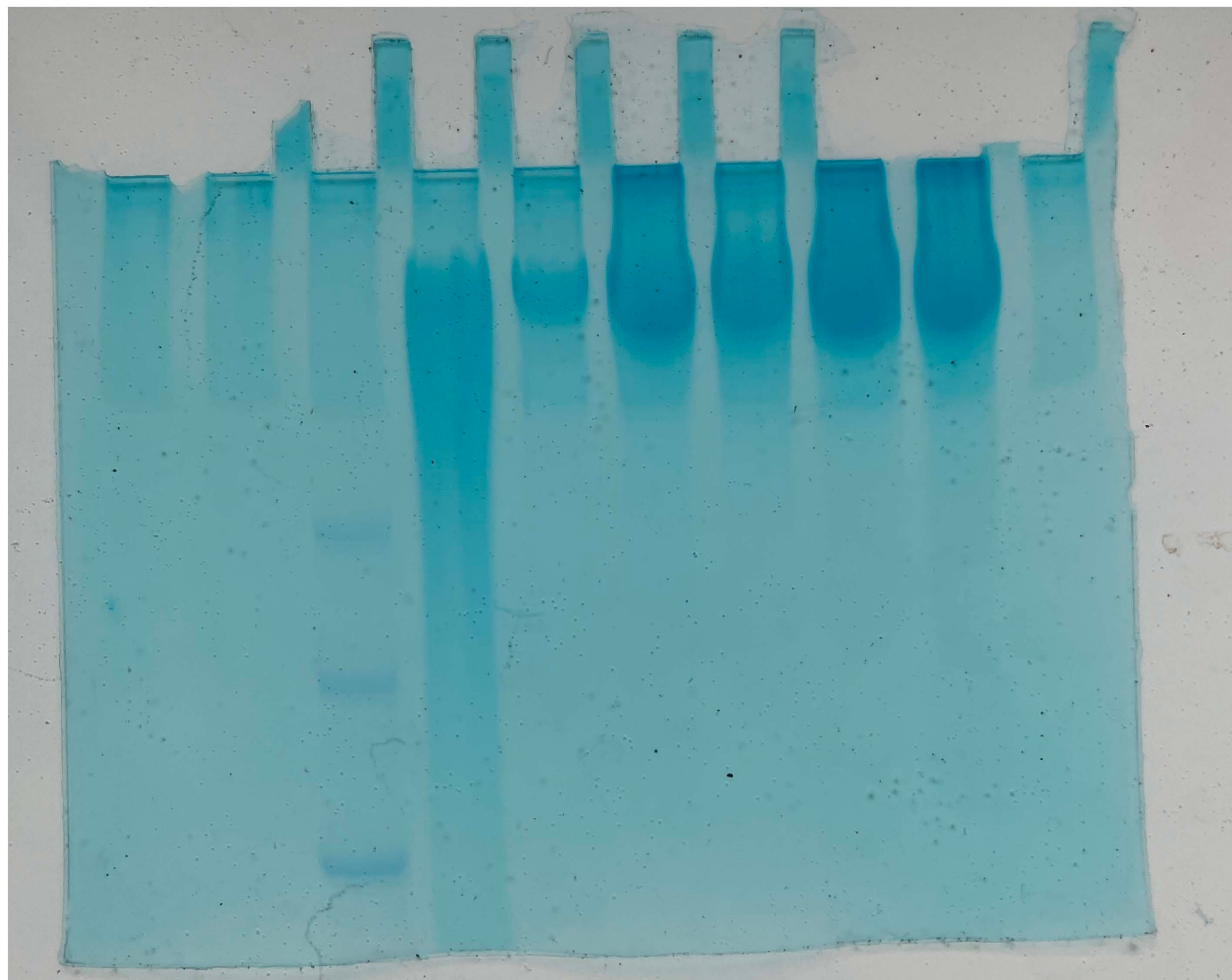

Fig 1b SDS-PAGE analysis of  $\gamma$ -PGA

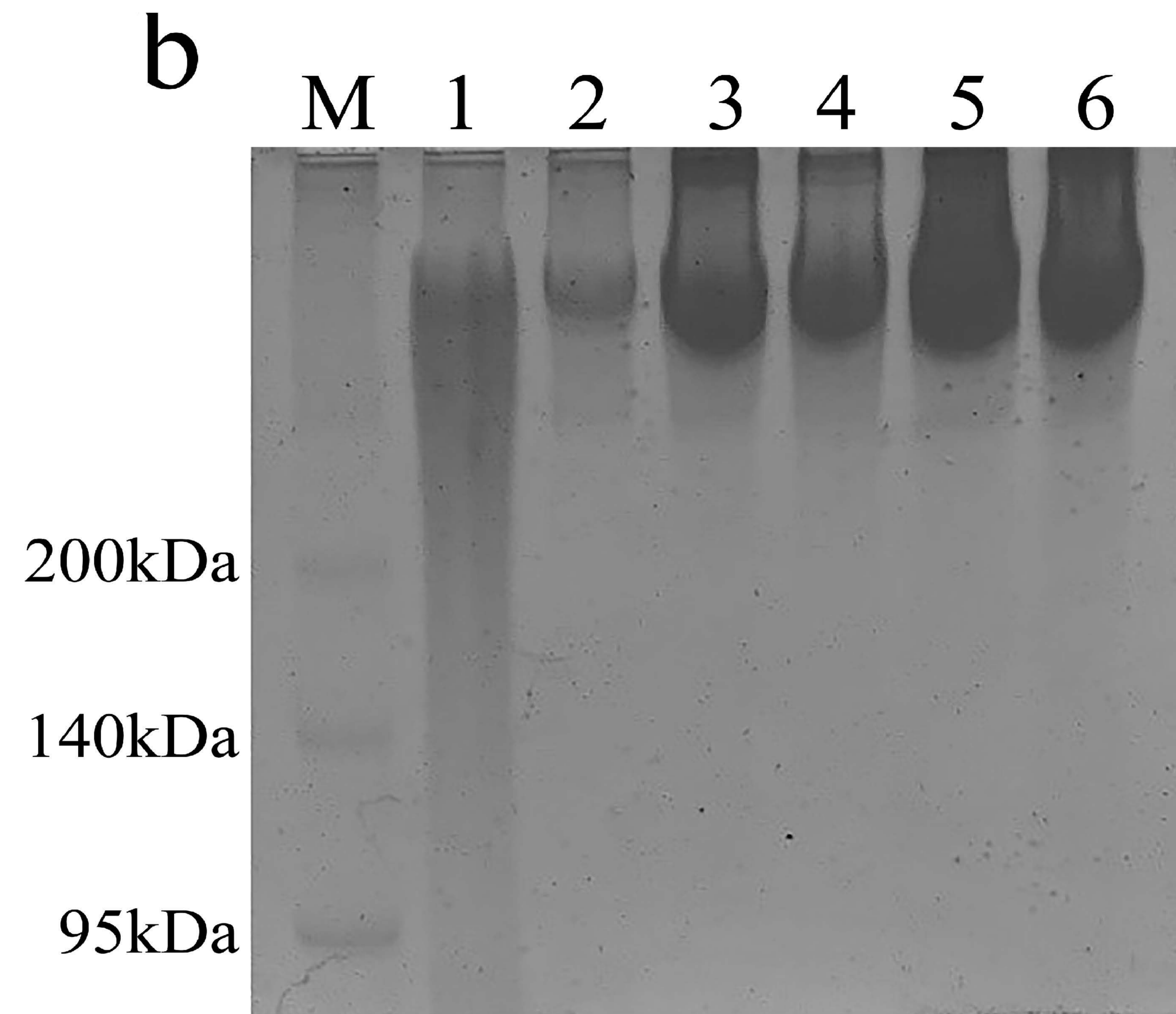

Fig 1b SDS-PAGE analysis of  $\gamma$ -PGA

Supplement: S1 Raw image — (PDF) [file pone.0310556.s003.pdf]
